# Interrogating the Behaviour of a Styryl Dye Interacting with a Mesoscopic 2D-MOF and Its Luminescent Vapochromic Sensing

**DOI:** 10.3390/ijms23010330

**Published:** 2021-12-28

**Authors:** Maria Rosaria di Nunzio, Mario Gutiérrez, José María Moreno, Avelino Corma, Urbano Díaz, Abderrazzak Douhal

**Affiliations:** 1 Departamento de Química Física, Facultad de Ciencias Ambientales y Bioquímica, and INAMOL, Universidad de Castilla-La Mancha, Av. Carlos III, s/n, 45071 Toledo, Spain; mrosaria.dinunzio@uclm.es (M.R.d.N.); mario.gutierrez@uclm.es (M.G.); 2 Instituto de Tecnología Química, Universitat Politécnica de Valéncia-Consejo Superior de Investigaciones Científicas (UPV-CSIC), Av. de los Naranjos, s/n, 46022 Valencia, Spain; josemmorenorodriguez@gmail.com (J.M.M.); acorma@itq.upv.es (A.C.); udiaz@itq.upv.es (U.D.)

**Keywords:** hybrid materials, host-guest interaction, aggregates, time-resolved spectroscopy, luminescent vapochromic sensing

## Abstract

In this contribution, we report on the solid-state-photodynamical properties and further applications of a low dimensional composite material composed by the luminescent trans-4-(dicyanomethylene)-2-methyl-6-(4-dimethylaminostyryl)-4H-pyran (DCM) dye interacting with a two-dimensional-metal organic framework (2D-MOF), Al-ITQ-HB. Three different samples with increasing concentration of DCM are synthesized and characterized. The broad UV-visible absorption spectra of the DCM/Al-ITQ-HB composites reflect the presence of different species of DCM molecules (monomers and aggregates). In contrast, the emission spectra are narrower and exhibit a bathochromic shift upon increasing the DCM concentration, in agreeance with the formation of adsorbed aggregates. Time-resolved picosecond (ps)-experiments reveal multi-exponential behaviors of the excited composites, further confirming the heterogeneous nature of the samples. Remarkably, DCM/Al-ITQ-HB fluorescence is sensitive to vapors of electron donor aromatic amine compounds like aniline, methylaniline, and benzylamine due to a H-bonding-induced electron transfer (ET) process from the analyte to the surface-adsorbed DCM. These findings bring new insights on the photobehavior of a well-known dye when interacting with a 2D-MOF and its possible application in sensing aniline derivatives.

## 1. Introduction

Across the last three decades, MOFs have emerged as materials with an extensive applicability in many fields of modern science and technology [1]. MOFs are crystalline materials generated by the self-assembly of metal ions or clusters with organic linkers through coordination bonds, which provides porous structures with large surface areas [2]. MOFs show great design versatility in terms of channel/pore volume, shape, and dimensions by an adequate selection and functionalization of the organic strut and the secondary building units (SBUs) [3]. Commonly, MOFs form three-dimensional (3D) structures through the assembly of the subunits in the three space directions. The network of these materials is considered to be relatively rigid, while weak interactions like H-bonds or π-π stacking may provide a certain flexibility to the system [4]. Additionally, the possibility of incorporating a wide range of guests in their porous structures offers a limitless variety of combinations, making them even more attractive for diverse applications like gas separation and storage, chemical sensing, bioimaging, drug (photo) delivery, optoelectronic devices, heterogeneous catalysis, and photocatalysis [5,6,7,8].

**Scheme 1:**
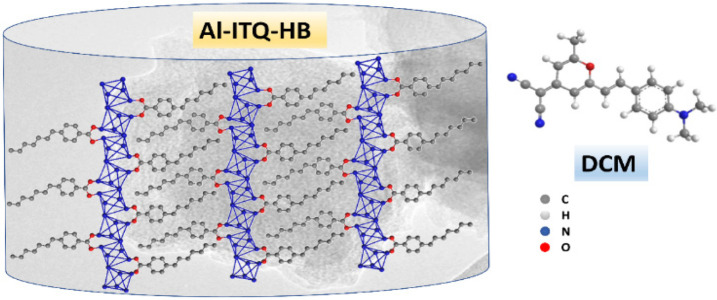
Schematic representation (not in scale) illustrating the Al-ITQ-HB framework together with the molecular structure of DCM.

On the other hand, over the last years, low dimensional 2D-MOFs have attracted the interest of the scientific community thanks to their unique qualities shown in important fields such as gas adsorption/separation [9,10], sensing [11,12], catalysis [13,14], and chemical release [15,16]. A 2D-MOF can be created by a single-layer assembly and the resulted sheet units provide 2D-structures having larger active surface areas, improving the diffusion of guest molecules [17,18,19]. Moreover, the absence of connection between the sheets generates free coordinating sites in the metal clusters acting as active sites in catalytic reactions [20]. One recent example of 2D-MOFs is Al-ITQ-HB (Scheme 1 and Scheme S1) based on the suitable assembling of 1D organic-inorganic structural sub-units (Scheme S2), which are constructed with octahedral aluminium clusters glued by 4-heptylbenzoic acid (HB) organic linkers. The resulting mesoscopic lamellar structure of this MOF can favour its interaction with organic molecules [21].

Research of MOF properties and host-guest interactions at the molecular level and at real-time are critical for upgrading the functionality of these hybrid compounds in fields such as photonics and photocatalysis. To this end, time-resolved laser-based techniques provide useful keys to describe the photodynamic scenario of MOFs comprising guest molecules [8]. *Trans*-4-(dicyanomethylene)-2-methyl-6-(4-dimethylaminostyryl)-4H-pyran (DCM, Scheme 1) is a well-known push-pull dye of wide use in the field of photophysics. It is characterized by a high fluorescence quantum yield and great stability [22]. DCM is largely used as a probe of local environment properties. Its photobehavior has been extensively investigated in a variety of solvents [23,24,25,26,27,28,29,30] and in presence of confined media such as micelles [31,32], lipid vesicles [33,34], microemulsion [35], human serum albumin (HSA) [36], polyvinyl carbazole (PVK) [37], polypeptide-surfactant aggregate [38], micro-/mesoporous materials [39,40], and MOFs [41]. All these investigations demonstrate that DCM experiences a photoinduced intramolecular charge transfer (ICT). This reaction occurs due to the presence of an electron donating group (dimethylamine; donor, D) and an electron withdrawing group (dicyanomethylene; acceptor, A) within the molecule structure. Upon electronic excitation, DCM firstly reaches the S_1_-locally excited (LE) state, whose electron density distribution and dipole moment are comparable to those of the ground-state, S_0_. In contrast, the CT species at the S_1_ state possesses a larger dipole moment as a result of a large charge separation upon electronic excitation (the difference between the ground- and excited-state dipole moments is around 20–26 D) [29,42]. It has been shown that excited DCM molecules undergo a twisting motion after charge separation, thus populating an excited twisted intramolecular charge transfer (TICT) state [29,43,44,45]. In a push-pull molecule, the TICT process can take place at different time constants, ranging from the femtosecond (fs) to the nanosecond (ns) regime (10^−13^–10^−8^ s), depending on the system under study and on the polarity of the medium which affects, among other factors, the height of the energy barrier for this process [28]. In apolar solvents like cyclohexane, the LE state of DCM is the only emitting one with a lifetime of 16 ps^28^ and no CT was observed at the excited-state [28]. By increasing the polarity of the medium, the decay times are longer (1.36 ns in MeOH and 2.24 ns in DMSO) and no emission from the S_1_-LE state was detected [23,25,26,27], indicating that the TICT process becomes faster. In most of the reports on DCM supported on solid surfaces, the LE emission has not been observed because the TICT reaction still remains ultrafast [31,32,33,34,35,36,37,38,39,40]. However, there are few studies where DCM deactivates from both LE and CT state such as when encapsulated within MCM41^39^ and into the pores of a Zr-naphthalene dicarboxylic acid (Zr-NDC) MOF [41]. In addition to the forementioned, the interaction of high concentration of DCM with different silica-based materials, such as faujasite-type zeolites (HY, NaX, and NaY) and MCM41, can lead to the formation of aggregated species [40]. For instance, it has been shown that H- and J-aggregates relax with lifetimes of 65–99 ps and 350–400 ps, respectively, while the monomers decay in the range of 2.44–3.9 ns [40]. Although the photodynamics of DCM molecules in solid 3D-porous materials is relatively well understood, its interaction with 2D-MOFs still remains unexplored. Therefore, a photophysical characterization of DCM interacting with 2D-MOFs will unravel the spectroscopic properties of its composites, paving the way for further implementations to developing efficient materials, as well as their possible applicability in different photonic technologies.

In this contribution, we explore the spectroscopic and photodynamical characteristics of three DCM/Al-ITQ-HB samples having increasing concentrations of DCM fluorophore (5 × 10^−6^, 1 × 10^−4^, and 1 × 10^−3^ M), aiming to unravel the photophysics of the composites and exploit it for vapochromic sensing applications. In particular, these characterizations are important for further use of these materials as luminescent probes of different electron-rich amine aromatic compounds, which induce a quenching of its fluorescence intensity. Therefore, the present study presents a deep photophysical characterization of DCM/Al-ITQ-HB materials and demonstrate their potential applicability in photonics.

## 2. Result and Discussion

### 2.1. Steady-State Observations

**Figure 1:**
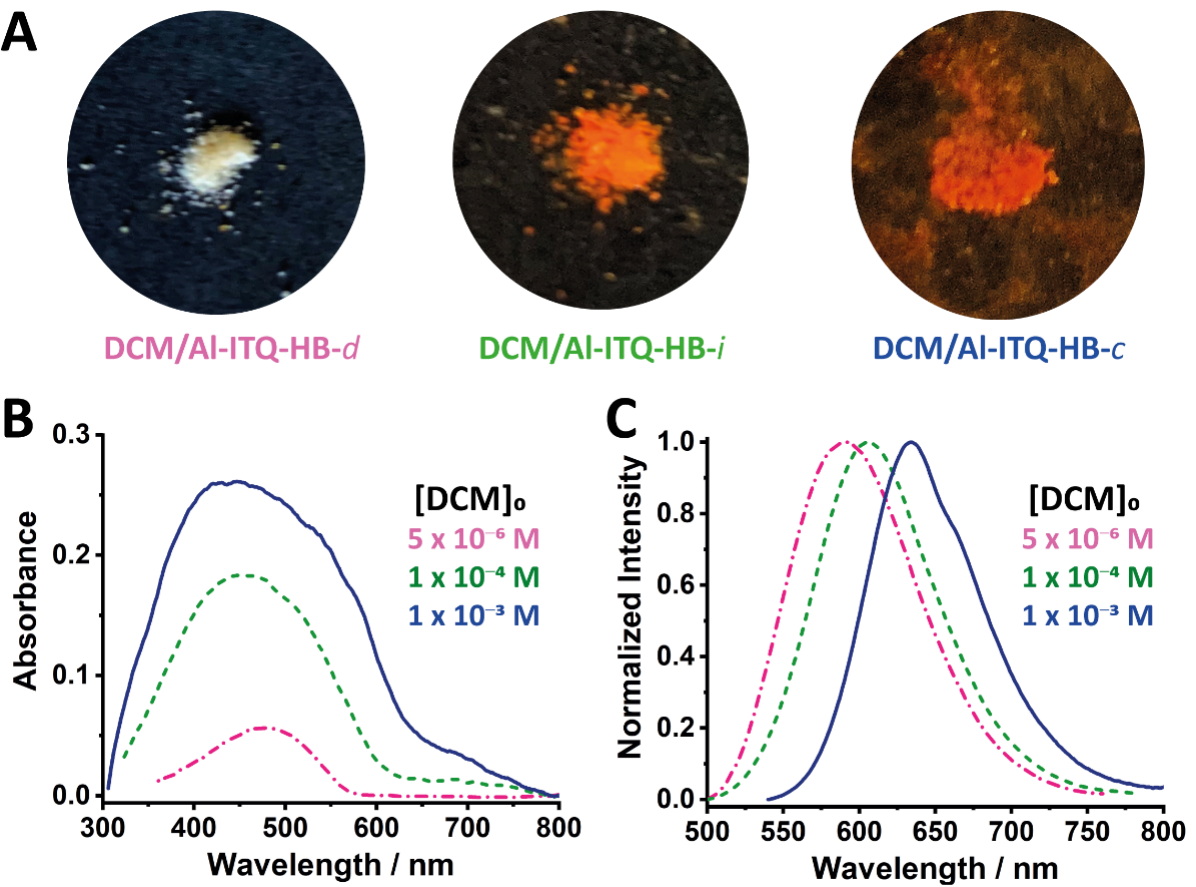
(**A**) Photographs of the hybrid composites (under daylight) containing increasing amounts of DCM. UV-visible absorption (**B**) and emission (**C**) spectra of DCM/Al-ITQ-HB in the solid-state at different dye concentrations: 1 × 10^−3^ M (blue solid line), 1 × 10^−4^ M (green dashed line), and 5 × 10^−6^ M (pink dashed-dotted line). Each absorption spectrum has been corrected by subtracting the absorption from pristine Al-ITQ-HB. For emission spectra, the excitation wavelength is 470 nm.

Three DCM/Al-ITQ-HB composites of different loadings were prepared using different concentrations of DCM dye: 5 × 10^−6^, 1 × 10^−4^, and 1 × 10^−3^ M. Considering the synthetic procedure of these hybrid composites (see Section 3) where the solvent is evaporated and the final hybrid composite sample is not washed, we can envisage that most of DCM molecules will be adsorbed on the MOF’s surface. Indeed, it has been described that a proper washing is necessary for removing the molecules adhered to the MOFs‘ surface, meaning that when the hybrid material is not properly washed, most of the molecules will be attached onto the MOF’s surface [46,47,48]. The colour of the obtained samples (DCM/Al-ITQ-HB-*d*, *d* = diluted; DCM/Al-ITQ-HB-*i, i* = intermediate; DCM/Al-ITQ-HB-*c*, *c* = concentrated) varies from light to vibrant orange when the initial dye concentration increases (Figure 1A). The absorption spectra of the investigated samples (obtained through the diffuse reflectance measurements) are shown in Figure 1B, while their emission ones are displayed in Figure 1C. Moreover, the spectra in Figure 1B have been corrected by subtracting the absorption of Al-ITQ-HB. Figure S1A shows the non-corrected spectra together with that of pristine Al-ITQ-HB, while Figure S1B exhibits the corrected, normalized ones of the composites compared with the spectrum of DCM in dichloromethane.

The absorption spectrum of the DCM/Al-ITQ-HB-*d* composite prepared with a concentration of [DCM]_0_ = 5 × 10^−6^ M exhibits a broad and structureless band with an intensity maximum at 478 nm (Figure 1B). This value is close to the one shown in dichloromethane solutions (466 nm) [30,40,49,50]. Therefore, we assign this band to the S_0_ (π) → S_1_ (π*) transition. Comparing the FWHM of the main absorption band of DCM in solution (4360 cm^−1^) and DCM/Al-ITQ-HB-*d* (6030 cm^−1^), we observe a band broadening upon interaction with the MOF, which suggests the existence of an heterogenous distribution of DCM molecules in the composites. This is also evidenced by the deviation from the normal Gaussian distribution of the absorption spectrum. On the other hand, the emission spectrum shows a narrow band (FWHM = 2810 cm^−1^) with its intensity maximum at 590 nm (Figure 1C). The smaller FWHM value of the emission spectrum compared to that obtained from the absorption one indicates the existence of population(s) of surface-adsorbed DCM molecules relaxing to S_0_ through fast non-radiative pathways. The spectral Stokes-shift ($\Delta {\overset{¯}{\nu }}_{abs-em}^{max}$ = 3970 cm^−1^) observed for the DCM/Al-ITQ-HB-*d* composite, being only slightly smaller compared to that ($\Delta {\overset{¯}{\nu }}_{abs-em}^{max}$ = 4510 cm^−1^) observed in a dichloromethane solution, indicates that an excited-state CT reaction occurs as well for the DCM molecules interacting with the MOF. To further characterize the nature of the DCM species adsorbed on the surface of Al-ITQ-HB, we have studied the photobehavior of two additional samples with increasing concentrations of DCM. The intermediate sample, DCM/Al-ITQ-HB-*i*, having an initial dye concentration of 1 × 10^−4^ M, shows an even broader absorption spectrum (FWHM = 9360 cm^−1^), with an intensity maximum at 456 nm (Figure 1B). This indicates a higher heterogeneity of the absorbing DCM species when the initial DCM concentration increases. A new band centred at 660 nm is also detected, being this band reminiscent to the absorption of bulk DCM in solid state, as shown in Figure S2. The emission of this composite is centred at 606 nm, with a Stokes shift of 5430 cm^−1^ (Figure 1C). This value is larger than that calculated for the diluted sample (DCM/Al-ITQ-HB-*d,* 3970 cm^−1^), revealing a clear dependence of the photobehavior of DCM/Al-ITQ-HB materials on the concentration of DCM. It is worth to note that, while the absorption spectrum becomes broader by increasing [DCM]_0_, the emission follows the opposite trend, appearing as a narrower band (FWHM = 2530 cm^−1^). This behaviour suggests that despite the larger number of ground-state species in the intermediate sample, the excited-state non-radiative deactivation paths become more efficient because of stronger interactions between the adsorbed DCM molecules. The highest concentrated sample, DCM/Al-ITQ-HB-*c* presents the broadest absorption spectrum (FWHM = 14,480 cm^−1^), with the most blue-shifted intensity maximum (448 nm, Figure 1B). The absorbance of the 660 nm-band is almost 4 times higher than that observed for DCM/Al-ITQ-HB-*i*, corroborating that this band arises from species behaving similarly to pristine DCM in solid state, and therefore will correspond to DCM species highly aggregated on the surface of the MOF. The emission spectrum is, on the other side, the one situated at the reddest region with its maximum at 634 nm and exhibiting a Stokes shift of 6550 cm^−1^ (Figure 1C). It presents the narrowest band (FWHM = 2125 cm^−1^), revealing that very high dye concentrations induce efficient non-radiative deactivation of the excited species, so that reducing their emission quantum yields.

All the aforementioned observations match well with the formation of DCM aggregates when increasing its concentration on the 2D-MOF. It is well known that several similar dyes can form H-(face-to-face) and J-(face-to-edge) aggregates, the two most common aggregate types (type I and II, respectively) [51]. According to the Kasha´s excitonic theory, the H- and J-aggregates absorb at higher and lower energies than the monomer, respectively, due to the different nature of the states involved in their transitions (S_0_ → S_2_ transition for H-aggregates, being the S_0_ → S_1_ one spin forbidden, and S_0_ → S_1_ transition for J-aggregates) [51,52,53,54,55]. One of the main differences between these two packing modes is the tilted angle (*θ*) between the chromophore’s plane and the packing direction, which is mayor or minor than the “magic angle” (*θ_M_* = 54.71°) for H- and J-aggregates, correspondingly. J-aggregates can also arrange forming one-dimensional (1D) columns (type III aggregates), where the offset angle (φ) between the long molecular axis is usually small [51]. Type III aggregates display the same spectroscopic features of J-aggregates. When φ is large enough to make the transition dipoles of neighbouring chromophores almost orthogonal to each other, X-aggregates (type IV) are formed [51]. For the later, the electronic coupling is so weak that both absorption and emission are very similar to those of the isolated monomers.

Based on these considerations, we attribute the large broadening in the absorption band of the highest concentrated DCM/Al-ITQ-HB materials to the formation of DCM H- and J-aggregates, respectively, without *a priori* excluding the possible additional formation of types III-and IV-aggregates. Moreover, the red-shift observed in the emission spectra of the highest concentrated samples further support this assumption, as H-aggregates are usually non-emissive (or weakly emissive), while the emission of J-aggregates emerges at lower energies than the monomers. On the other hand, the emission spectrum of the most diluted sample (DCM/Al-ITQ-HB-*d*) resembles to that observed for DCM in dichloromethane solution (Figure S1C), indicating that in this composite the DCM molecules are mainly in the form of adsorbed monomers.

To shed more light on the spectroscopic properties of these DCM aggregates, we have deconvoluted (assuming a Gaussian shape of the absorption band) the absorption spectra of all the samples. Figure S3 shows a deconvolution analysis of the spectra, while Table S1 reports the obtained results. For all the studied concentrations, there are at least three different populations co-existing at S_0_, and which absorption intensity maxima are at around 395, 470, and 528–538 nm. Therefore, and based on our previous explanations, we identify the absorbing species as H-aggregates, monomers, and J-aggregates, respectively. For DCM/Al-ITQ-HB-*i* and DCM/Al-ITQ-HB-*c* samples, a fourth band centred at ~660 nm (previously attributed to neat solid DCM) was required for an accurate fit of the spectrum. Table S1 also shows the absorbance value of each species for the studied samples. For the aggregates, the area of the spectra increases (from 18 to 32% for H-aggregates; and from 15 to 41% for J aggregates) with DCM concentration, consistently with the fact that the number of molecules adsorbed on the MOF’s surface increase, and therefore, the formation of aggregates must grow up as well. The increase of aggregates population with the concentration was also estimated by calculating the ratios between the integral areas of monomers and H-aggregates (R_1_ = M/H), monomers and J-aggregates (R_2_ = M/J), and H- and J-aggregates (R_3_ = H/J), respectively (Table S1). We found that increasing the initial dye concentration, R_1_ and R_2_ decrease by 5 and 9 times, respectively, indicating that aggregates are favoured with respect to monomers, and interestingly, J-aggregates tend to predominate over H-aggregates, as also supported by the decrease of R_3_ at the highest dye concentration.

To get further information on the absorbing and emitting species, the fluorescence excitation spectra of the DCM/Al-ITQ-HB composites are shown in Figures S4–S6 and compared to the corresponding absorption spectra. The comparison reveals the presence of at least two different ground-state species depending on the observation wavelength. At longer observation wavelengths (690 nm), the excitation spectra are red-shifted, while the spectra are blue-shifted when gating at shorter wavelengths (580 nm). This behaviour agrees with the presence of monomers and J-aggregates, further supporting the above discussion. Additionally, neither the band of the H-aggregates nor the 660 nm-peak (attributed to neat solid DCM) are detected in the excitation spectra of all DCM/Al-ITQ-HB compounds, further evidencing that these species are very weakly emissive (Figures S4–S6). Indeed, when the concentration of DCM dye increases, the molecules become to aggregate, decreasing in this way the emission intensity of the composite (Figure S7).

### 2.2. Time-Resolved Emission Measurements

**Figure 2:**
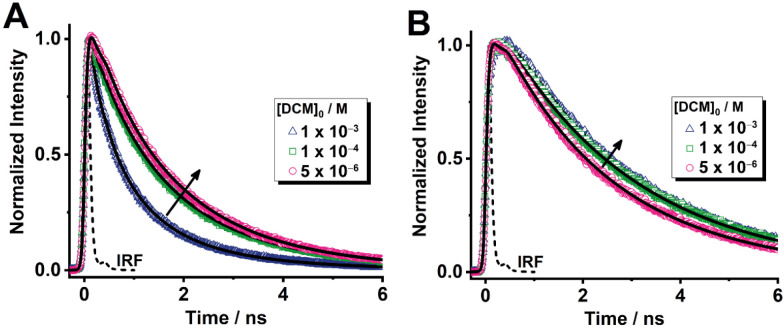
Normalized (to the maximum intensity) magic-angle emission decays of DCM/Al-ITQ-HB at different initial dye concentrations: 1 × 10^−3^ M (blue triangles), 1 × 10^−4^ M (green squares), and 5 × 10^−6^ M (pink circles) upon excitation at 470 nm and observing at 550–575 (**A**) and 700–750 (**B**) nm. The solid lines are from the best-fit using a multi-exponential function. IRF (black dashed line) is the instrumental response function.

To get insight on the photodynamics of the DCM/Al-ITQ-HB composites, we have performed ps time-resolved emission experiments. The samples were excited at three different wavelengths to get a full insight on the photobehavior of the different species adsorbed on the Al-ITQ-HB’s surface. Figure 2 shows the emission decays of DCM/Al-ITQ-HB composites excited at 470 nm and recording at different wavelengths of the emission spectrum, i.e., in the green (A) and red (B) parts. Figures S8 and S9 show additional emission decays upon excitation at 371 and 510 nm, respectively. Table 1 gives the time constants (*τ*_i_), normalized (to 100) pre-exponential factors (*a*_i_), and relative contributions (*c*_i_) obtained using a global fit.

**Scheme 2:**
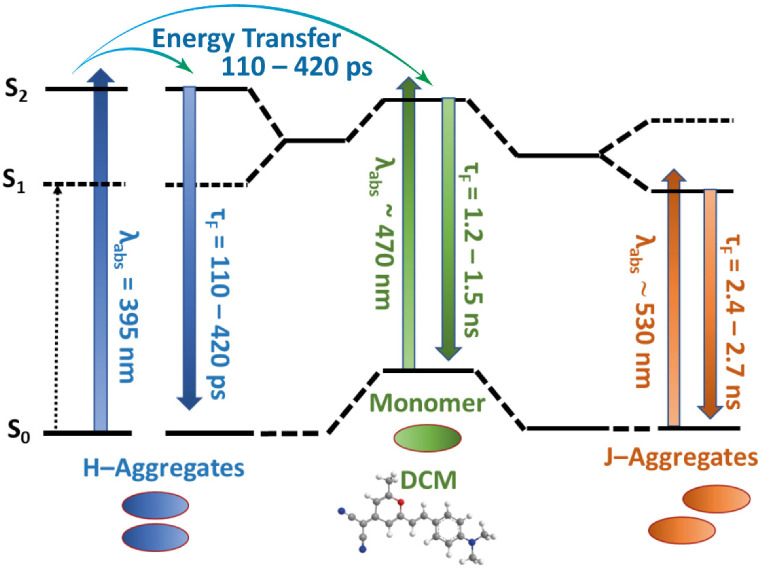
Illustration (not in scale) of the energetic levels of DCM monomers, H- and J-aggregates. The dotted arrow corresponds to a forbidden transition, while the filled ones reflect the absorption and emission transitions together with the corresponding wavelengths and lifetimes. The scheme also illustrates the EnT happening between H-aggregates and from H-aggregates to monomers.

To begin with, we discuss the results upon excitation at 470 nm: at this wavelength, monomers present the highest absorbance intensity compared to those of H- and J-aggregates (Figure 1B, and the above discussion in Section 2.1). All the decays were well fitted to a three-exponential function with lifetimes of 180–420 ps (*τ*_1_), 1.4–1.5 ns (*τ*_2_), and 2.5–2.7 ns (*τ*_3_). *τ*_1_ and *τ*_2_ contribute mainly in the green part of the spectrum, while *τ*_3_ predominates in the reddest spectral region. In the following, we explain the assignment made for each component. Firstly, *τ*_2_ component is reminiscent to that observed for DCM monomers in solutions (1.13–2.25 ns, depending on the nature of the solvent) [40,56], so it can be attributable to the emission lifetime of the monomeric species. Interacting with the MOF surface. *τ*_3_ component contributing more to the signal in the reddest spectral region, may therefore be assigned to the emission of J-aggregates. According to the excitonic theory, the emission of H-aggregates is a forbidden transition, so their lifetime much be much shorter than that of J-aggregates. Thus, even it would be possible to detect the emission of H-aggregates using TCSPC technique, in general their emission will be not easily observable in a stationary emission spectrum. Based on these considerations, we assign *τ*_1_ to the H-aggregates emission, which mainly contributes at the highest energies and is characterized by a shorter lifetime. Moreover, for all the investigated concentrations, *τ*_1_ appears as a decay in the green part (from 525 to 600 nm), while it rises in the reddest region of the spectrum (from 600 nm, Table 1, and Figure S10A). This is an indication of a dynamical process which involves excited-state species. Given the long duration of this process (110 to 420 ps), the ICT can be discarded, since this is an ultra-fast phenomenon occurring within times less than the resolution of our TCSPC system (about 20 ps). Taking in mind the synthetic method of this system, and the presence of monomers and aggregates in the DCM/Al-ITQ-HB composites, the surface-adsorbed DCM molecules which are in close proximity one each other may experience an EnT process. The possibility of an EnT is further supported by the strong overlap between the broad absorption and emission spectra observed for all the DCM/Al-ITQ-HB materials (Figure S11). Therefore, the shorter *τ*_1_-component, which is manifested in the signals as decay at shorter observation wavelengths, and rise at longer ones, can be a combination reflecting the lifetime of H-aggregates and the dynamics of the EnT process. Based on the spectral overlap and the change of the relative contributions of the emitters in the signal, we suggest that this EnT event might occur as a homoEnT (within H-aggregates) or from H-aggregates to monomers, as depicted in Scheme 2. Indeed, aggregate-to-aggregate EnT has been observed in a vast number of molecular packing geometries [55,57].

To further elucidate which is the most plausible EnT mechanism, we have explored the photophysics of these materials upon increasing DCM concentration, and exciting at different wavelengths where it is possible to selectively photoexcite the ground state species. Analysing the effect of the initial DCM concentration on the emission decays, Table 1 shows that *τ*_1_ increases from 180 to 420 ps when [DCM]_0_ grows from 5 × 10^−6^ to 1 × 10^−3^ M, whereas *τ*_2_ and *τ*_3_ values do not practically change. On the other side, the relative contributions of the three components are clearly affected by the initial DCM concentration. In particular, going from less to more concentrated samples, the contribution of the short component (*c*_1_) increases, indicating that the population of H-aggregates become more abundant when increasing the initial dye concentration, in full agreement with results of the deconvolution analysis of the absorption spectra. On the other hand, *c*_3_ shows its highest values at lower spectral energies. For *c*_2_, it remains pretty constant from [DCM]_0_ = 5 × 10^−6^ to 1 × 10^−4^ M, while at the highest dye concentration it slightly decreases. Its pre-exponential factor (*a*_2_) reaches the maximum value around 500–575 nm. These observations further confirm our assignment of H-aggregates, monomers, and J-aggregates to *τ*_1_, *τ*_2_, and *τ*_3_, respectively. Considering Figure S11, the largest spectral overlap is observed for the DCM/Al-ITQ-HB-*c* material, which generally should be reflected in a higher EnT probability (shorter lifetime). However, we observed the opposite tendency: the decay of the DCM/Al-ITQ-HB-*c* sample displays the slowest EnT process (420 ps) compared to the DCM/Al-ITQ-HB-*d* one (180 ps). This suggests that an EnT between H-aggregates might occur by a multistep hopping mechanism, whose rate constant increases with the dye concentration due to the higher number of D and A species leading to longer hopping distances. This experimental evidence further suggests that the most probable EnT mechanism is that involving H-aggregates.

Pumping the samples at a shorter wavelength (371 nm), we are mainly exciting H-aggregates, a weak population of monomers, and a smaller one of J-aggregates. Under these conditions, we also used a three exponential function to fit the decays, and the obtained lifetimes are: 110–170 ps, 1.2–1.3 ns, and 2.6 ns (Table 1). *τ*_1_ almost does not vary with the concentration, being the lifetimes values within the estimated uncertainty (±50 ps). The intermediate (*τ*_2_) and longest (*τ*_3_) components are practically not affected by the initial dye concentration. Similar to what observed by exciting at 470 nm, *τ*_1_ and *τ*_2_ predominate mostly in the green spectral region, whilst *τ*_3_ shows its maximum contribution at lower emission energies. A risetime of 110–170 ps (a value similar to that of the shortest decaying component) is also observed from 630 nm (Table 1 and Figure S10B), corroborating the previously discussed EnT processes. Increasing the initial DCM concentration, the contribution of *τ*_1_ and *τ*_2_ (*c*_1_ and *c*_2_) increases, where *c*_3_ decreases consistently with the 470 nm-excitation results. The excitation at 371 nm makes the EnT time constant shorter compared to those found upon excitation at 470 nm. This difference can be explained in terms of faster EnT at higher excitation energy bringing the system to higher vibronic energy levels which promote the EnT process (faster dynamics) [46].

Finally, irradiating at 510 nm (lower excess energy at S_1_) leads to the excitation of monomers (the major fraction) and J-aggregates, but not H-aggregates. Under these experimental conditions, we observed three-exponential decays for all the interrogated samples (Table S2). The time constants are: 160–170 ps (*τ*_1_), 1.3–1.4 ns (*τ*_2_), and 2.4–2.7 ns (*τ*_3_). The intermediate (*τ*_2_) and longest (*τ*_3_) components correspond to the emission from monomers and J-aggregates, respectively, whereas *τ*_1_ is assigned to excited neat solid DCM adsorbed on the MOF surface. The contribution of *τ*_1_ increases with the initial dye concentration, consistently with its designation (Table S2). Interestingly, we did not observe any rising component (Figure S10C), which further confirms that the EnT process is not occurring either from monomers or J-aggregates. These observations, upon changing the initial concentration of DCM and the excitation wavelength, support the assignment that the 110–420 ps component involves an EnT process from H-aggregates (Scheme 2).

**Figure 3:**
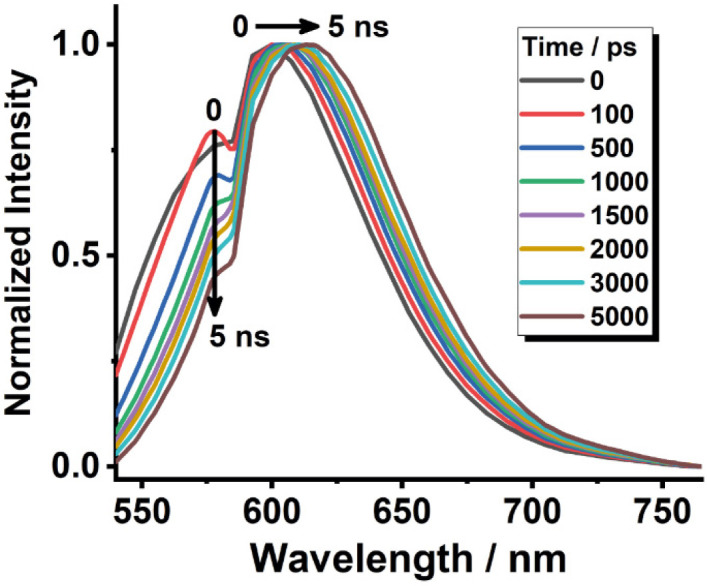
Normalized (to the maximum intensity) TRES of DCM/Al-ITQ-HB using initial dye concentration of 1 × 10^−3^ M upon excitation at 470 nm and gating at the indicated delay times.

The fluorescence behaviour of the composite upon excitation at 470 nm was further studied by recording time-resolved emission spectra (TRES). In Figure 3, we observe a short-living emission at 540–605 nm and a longer one at 605−750 nm. The blue-shifted short-living emission band, nicely match with the emission of H-aggregates. On the other hand, at longer gating times (5 ns), both the shape and position of the TRES are changed with respect to time zero and they are pretty similar to the steady-state ones (Figure 1B). These observations agree with the photobehavior of DCM/Al-ITQ-HB-*c* explained above and shown in Figure 2 and Table 1.

### 2.3. Luminescence Vapochromic Sensing

The detection of chemical compounds in the vapor phase has attracted much attention owing to the potential applicability of vapochromic sensors in the monitoring of the air quality in industrial or domestic environments, or the detection and control of medical conditions, such as diabetes [58,59,60]. Special attention has been paid to the detection of harmful gases commonly employed in industrial processes, where a continuous tracking is needed to guarantee a healthy work environment. One interesting example is the aniline (AN) and its derivatives: methylaniline (MAN), dimethylaniline (DMA) or benzylamine (BAM); which are toxic compounds employed in several industrial processes, like the production of dyes and textile, rubber assistants, raw material for pesticides, etc. Consequently, there exist a necessity to detect these compounds in the vapor phase to prevent their exposure to the human being.

**Figure 4:**
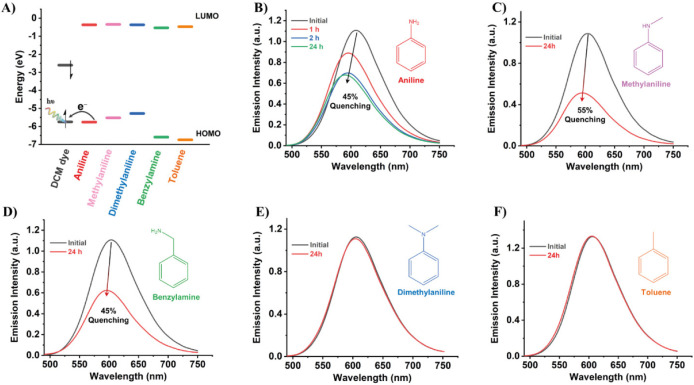
(**A**) Schematic representation of the HOMO-LUMO energy levels (in gas phase) for the DCM dye and the aromatic compounds used for the quenching test (aniline, methylaniline, dimethylaniline, benzylamine, and toluene). (**B**–**F**) Emission spectra of DCM/Al-ITQ-HB ([DCM]_0_ = 1 × 10^−4^ M) in the absence and presence of aniline (**B**), methylaniline (**C**), benzylamine (**D**), dimethylaniline (**E**), and toluene (**F**) atmospheres. For (**B**–**D**) the percentage of emission quenching is displayed in the figure.

From a chemical point of view, these aniline derivatives are generally good electron donor systems. Having this in mind, we have explored which conditions favour electron transfer (ET) from these compounds to DCM/Al-ITQ-HB ([DCM]_0_ = 1 × 10^−4^ M) composites. The ET phenomenon usually induces a quenching of the emission intensity and/or a shifting of the spectral position (change of the emission color) of the electron acceptor [61]. To investigate this possibility, we have firstly optimized the geometry and calculated the HOMO-LUMO energy levels of DCM dye, toluene (TOL), and the aniline derivatives using density functional theory (DFT) and applying the B3LYP/6-311^++^G(d,p) method. From these theoretical results, we can envisage that ET may occur from AN, MAN and DMA, whose HOMO energy levels are higher than that of DCM (Figure 4A). However, and following our calculations, this ET could not take place either from BAM or TOL. To experimentally examine these possibilities, we have exposed DCM/Al-ITQ-HB composites (in a closed desiccator) to a saturated atmospheres of AN, MAN, DMA, BAM, and TOL.

**Scheme 3:**
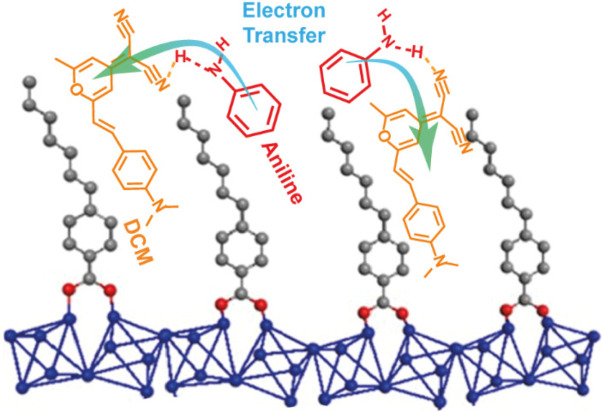
Schematic representation (not in scale) of the emission quenching mechanism of DCM/Al-ITQ-HB in presence of aniline.

As expected, the prolonged exposition of DCM/Al-ITQ-HB to AN vapor produces a quenching of its emission intensity and a blue-shift of its spectral position (Figure 4B), in agreement with the occurrence of an ET event from AN to the composite. In these experiments, we have recorded the emission spectrum at different times of exposure to AN to how the emission intensity decreases. Remarkably, we observed that there is almost no change between 2 and 24 h of exposition (~42 and 45% of quenching, respectively, Figure 4B), which reflects the relatively quick response of our material to the presence of vapors of AN. Similarly, the exposure to vapors of MAN induced a strong quenching (55%) of the emission intensity of DCM/Al-ITQ-HB and a small shift to shorter wavelengths (Figure 4C). On the other hand, and as predicted, exposure to TOL atmosphere does not produce any change in the emission spectrum of the MOF composite (Figure 4F). However, and very surprisingly, the response of DCM/Al-ITQ-HB to the presence of BAM and DMA is clearly unexpected. While the HOMO energy of DMA is higher than that of DCM (Figure 4A), and therefore a more pronounced emission quenching should be detected, that of BAM (lower than that of DCM) should prevent any ET to the MOF composite (Figure 4A). However, what we observed is that the exposition to BAM induced a large quenching (45%) of the emission intensity alongside with a spectral shift to higher energies (Figure 4D), while DMA did not alter the emission spectrum of DCM/Al-ITQ-HB (Figure 4E). This unpredicted behavior could be explained attending to the chemical composition of the aniline derivatives. The common component of the analytes that quench and shift the emission of DCM/Al-ITQ-HB is the presence of a hydrogen (H) atom on the electron rich nitrogen site prone to interact through H-bonds with the DCM. Therefore, we propose that the sensing mechanism rely on an ET from the aniline derivatives induced by H-bonding interactions with adsorbed DCM molecules (Scheme 3).

Indeed, it has been previously demonstrated that H-bond interactions can favor ET processes. For instance, a Zr-based MOF has shown an outstanding response to trinitrophenol (explosive molecule), contrary to other similar nitroaromatics, due to an ET process facilitated by H-bonding interactions [62]. These interactions may simultaneously alter the HOMO energy levels of adsorbed DCM and of the analytes, making the ET process more or less efficient. Taking into account the large emission quenching in presence of BAM, contrary to what is expected from the position of its HOMO energy level relatively to that of DCM (in gas phase), we suggest that DCM molecules interacting with the MOF surface have lowered their HOMO energy level more than that of BAM. Hence, for the present system under study, there should exist some degree of H-bond interactions between adsorbed DCM and the analyte molecules to induce an efficient ET, being this the reason behind the lack of response to the presence of DMA.

## 3. Materials and Methods

Anhydrous dichloromethane (DCM, spectroscopic grade ≥ 99.8%), aniline (99.5%), *N*-methylaniline (98%), *N,N*-dimethylaniline (99%), benzylamine (99%), and toluene (anhydrous, 99.8%) were purchased from Sigma-Aldrich and used without further purification.

### 3.1. Synthesis and Characterization of the Materials

The Al-ITQ-HB MOF was synthesized followed the method described elsewhere [21]. The characterization of Al-ITQ-HB and related composites (powder X-ray diffraction, PXRD; thermogravimetric analysis, TGA; differential thermal analysis, DTA; transmission electron microscopy, TEM; cross-polarization magic-angle spinning carbon-13 nuclear magnetic resonance, CP/MAS NMR ^13^C) is reported in Supplementary Material (Figures S12–S17, Table S3, and Schemes S1 and S2).

The PXRD pattern of Al-ITQ-HB (HB: heptylbenzoate chain) MOF is reported in Figure S12. In particular, Al-ITQ-HB material exhibited a mesoscopic phase when monoalkylcarboxylate linkers were used as structural spacers, such as heptylbenzoate moieties (HB). More in detail, PXRD pattern of the hybrid material showed one intense (100) diffraction band at low 2θ angles range at 35 Å. This characteristic band was associated to porous materials with short-range ordering and with internal mesoporous cavities (Figure S12). Scheme S1 shows the mesoscopic structuration level of hybrid Al-ITQ-HB materials where association of 1D structural sub-units can be appreciated.

DCM was adsorbed on the MOF’s surface using the following method. First, a solution of DCM with a known concentration (5 × 10^−6^, 1 × 10^−4^, or 1 × 10^−3^ M) in dichloromethane was prepared. Subsequently, 1 or 2 mL of the DCM/dichloromethane solution were added to 50 or 100 mg of MOF and the mixture was stirred for 24 h at room temperature. Finally, the excess of solvent was eliminated by evaporation. The resulting deep orange composite, DCM/Al-ITQ-HB, resulted in DCM molecules mostly adsorbed on the MOF’s surface.

### 3.2. Experimental Procedures

#### 3.2.1. Luminescent Vapochromic Experiments

For the luminescent vapochromic sensing, the DCM/Al-ITQ-HB material was exposed to saturated atmospheres of the aromatic amine molecules. To this end, the composite material was placed in a desiccator together with a beaker containing 15 mL of the corresponding aromatic amine. Then, we applied ultrahigh vacuum on the desiccator for 5 min in order to evaporate the aromatic amine and generate a saturated atmosphere. Note that the MOF composite and the beaker were separated enough to avoid than any drop of the liquid could contact the solid powder. The sample and the beaker were kept in the closed desiccator for different periods of time ranging from 1 h to 24 h.

#### 3.2.2. Steady-State Spectroscopic and Time-Resolved Measurements

Steady-state UV-visible absorption and DT spectra were performed using a Jasco V-670 double-beam spectrophotometer provided with a 60-mm integrating sphere (ISN-723). Emission and excitation spectra were carried out with a Fluoromax-4 (Jobin-Yvone) spectrofluorometer. The emission decays were recorded with a ps TCSPC spectrophotometer (FluoTime 200, PicoQuant) previously described [63]. The fluorescence signal was collected at *θ_M_* = 54.71° and observed at a 90° angle with respect to the excitation beam at distinct emission wavelengths. We excited the samples using either 40-ps pulsed diode lasers focused at 371 or 470 nm (~1 mW, 40 MHz repetition rate) or a fs optical parametric oscillator (Inspire Auto 100) pumped by 1020 nm pulses (90 fs, 2.5 W, 80 MHz) from a Ti:sapphire oscillator (MaiTai HP, Spectra Physics) to generate the excitation beam at 510 nm (~50 µW). The instrument response function (IRF) is ~70 and ~35 ps for the diode laser and optical parametric oscillator, respectively. The experimental decays were assessed by the FluoFit software set of PicoQuant. Exponential decay functions were convoluted to IRF to fit the data. The shortest component which can be determined after a convolution process is 15 ps. The number of exponentials were prudently selected based on the reduced χ^2^ values, which were always below 1.2, and the residuals distributions. The estimated uncertainty of the time constants, considering the errors from the experiments as well as those arising from the multi-exponential fit of the signals, was ≤20%. The temperature in the laboratory during the experiments was 295 K.

## 4. Conclusions

In this work, we have disentangled the spectral and photophysical properties of three DCM/Al-ITQ-HB materials having increasing concentrations of DCM dye: 5 × 10^−6^, 1 × 10^−4^, and 1 × 10^−3^ M. All the studied samples show broad absorption bands in accordance with the presence of different absorbing species at the ground state. Moreover, the narrow profiles of the emission spectra indicate fast non-radiative deactivation for some excited species, reflecting the formation of aggregates. The experimental decays of the studied DCM/Al-ITQ-HB complexes are fitted using tri-exponential functions giving lifetimes of 110–420 ps, 1.2–1.5 ns, and 2.4–2.7 ns. The shortest component is assigned to a hopping mediated-EnT involving mainly H-aggregates, while the longest time represents the emission from J-aggregates. The middle time corresponds to DCM monomers. The prolonged exposition of DCM/Al-ITQ-HB (1 × 10^−4^ M) to aniline and methylaniline vapor induced a quenching of its emission intensity and a blue-shift of its spectral position. However, while the exposition to aniline induced a large quenching (45%) of the emission intensity concomitant with a shift to higher energies, the dimethylaniline did not affect the emission intensities of DCM/Al-ITQ-HB. These results suggest the relevance of an H-bonding-induced ET process between the analyte and the surface-adsorbed DCM molecule. These findings present new insights on the behaviour of DCM/Al-ITQ-HB composites, focusing on the possibility to tune the photobehavior of the interacting molecular probes. The information that this work brings would be of interest for the design of new materials for different applications like vapor photosensing.

## Figures and Tables

**Table 1:** Values of time constants (*τ*_i_), normalized (to 100) pre-exponential factors (*a*_i_), and contributions (*c*_i_) obtained from the fit of the emission decays of DCM interacting with Al-ITQ-HB MOF in the solid-state at different initial dye concentrations: 1 × 10^−3^ M, 1 × 10^−4^ M, and 5 × 10^−6^ M. The excitation wavelengths are: 470 and 371 nm, while the observation wavelengths are indicated in the Table. The estimated uncertainty of the time constants, considering the errors from the experiments as well as those arising from the multi-exponential fit of the signals, was between 15 and 20%.

**Sample/Exc = 470 nm**	**λ_obs_/nm**	***τ*_1_/ps (±50)**	***a*_1_**	***c*_1_**	***τ*_2_/ns (±0.2)**	***a*_2_**	***c*_2_**	***τ*_3_/ns (±0.3)**	***a*_3_**	***c*_3_**
DCM/ Al-ITQ-HB (c_0_ = 1 × 10^−3^ M)	550	420	56	25	1.5	41	65	2.7	3	10
	575		28	8		54	58		18	34
	600		8	2		49	38		43	60
	630		–100	–100		29	19		71	81
	660		–100	–100		12	7		88	93
	700		–100	–100		5	3		95	97
	720		–100	–100		5	3		95	97
DCM /Al-ITQ-HB (c_0_ = 1 × 10^−4^ M)	525	270	37	9	1.5	58	77	2.7	5	14
	550		19	3		63	64		18	33
	575		7	1		54	43		39	56
	600		–100	–100		36	24		64	76
	630		–100	–100		11	6		89	94
	660		–100	–100		2	1		98	99
	700		–100	–100		2	1		98	99
DCM/ Al-ITQ-HB (c_0_ = 5 × 10^−6^ M)	525	180	32	5	1.4	55	67	2.5	13	28
	550		15	2		56	51		29	47
	575		7	1		46	35		47	64
	600		–100	–100		34	21		66	79
	630		–100	–100		18	10		82	90
	660		–100	–100		11	4		89	96
	700		–100	–100		11	4		89	96
**Sample/Exc = 371 nm**	**λ_obs/_nm**	***τ*_1_/ps (±50)**	***a*_1_**	***c*_1_**	***τ*_2_/ns (±0.2)**	***a*_2_**	***c*_2_**	***τ*_3_/ns (±0.3)**	***a*_3_**	***c*_3_**
DCM/ Al-ITQ-HB (c_0_ = 1 × 10^−3^ M)	550	170	53	12	1.2	41	66	2.6	6	22
	575		30	4		49	51		21	45
	700		–100	–100		2	1		98	99
	720		–100	–100		1	1		99	99
DCM/ Al-ITQ-HB (c_0_ = 1 × 10^−4^ M)	525	160	49	9	1.3	43	65	2.6	8	26
	550		31	4		47	49		22	47
	660		–100	–100		1	1		99	99
	700		–100	–100		1	1		99	99
DCM/ Al-ITQ-HB (c_0_ = 5 × 10^−6^ M)	525	110	40	4	1.2	40	45	2.6	20	51
	550		20	2		43	34		37	64
	630		2	1		11	5		87	94
	660		–100	–100		8	4		92	96

## Data Availability

Exclude this statement.

## Supplementary Materials

The following are available online at https://www.mdpi.com/article/10.3390/ijms23010330/s1.

## Abbreviations

DCM	*trans*-4-(Dicyanomethylene)-2-Methyl-6-(4-Dimethylaminostyryl)-4H-Pyran
2D	Two-Dimensional
MOF	Metal Organic Frameworks
UV	Ultraviolet
ps	Picosecond
EnT	Energy Transfer
CT	Charge Transfer
SBU	Secondary Building Unit
3D	Three-Dimensional
HB	4-Heptylbenzoic Acid
HSA	Human Serum Albumin
PVK	Polyvinyl Carbazole
ICT	Intramolecular Charge Transfer
D	Donor
A	Acceptor
LE	Locally Excited
TICT	Twisted Intramolecular Charge Transfer
ns	Nanosecond
fs	Femtosecond
MeOH	Methanol
DMSO	Dimethyl Sulfoxide
MCM41	Mobil Catalytic Materials of number 41
NDC	Naphthalene Dicarboxylic Acid
FWHM	Full Width at Half Maximum
1D	One-Dimensional
TCSPC	Time-Correlated Single-Photon Counting
TRES	Time-Resolved Emission Spectra
AN	Aniline
MAN	Methylaniline
DMA	Dimethylaniline
BAM	Benzylamine
TOL	Toluene
HOMO	Highest Occupied Molecular Orbital
LUMO	Lowest Unoccupied Molecular Orbital
DFT	Density Functional Theory
ET	Electron Transfer
PXRD	Power X-ray Diffraction
TGA	Thermogravimetric Analysis
DTA	Differential Thermal Analysis
TEM	Transmission Electron Microscopy
CP/MAS NMR ^13^C	Cross-Polarization Magic-Angle Spinning Carbon-13 Nuclear Magnetic Resonance
IRF	Instrument Response Function
