# Supplementary material for: Interrogating the Behaviour of a Styryl Dye Interacting with a Mesoscopic 2D-MOF and Its Luminescent Vapochromic Sensing
Source: Int J Mol Sci. 2021 Dec 28;23(1):330. doi: 10.3390/ijms23010330 (PMC8745538; doi:10.3390/ijms23010330)
Supplement: Supplementary file 1 [file ijms-23-00330-s001.zip › ijms-1429005-supplementary.pdf]

Supplementary Materials

for

Interrogating the Behaviour of a Styryl Dye Interacting with a Mesoscopic 2D-MOF and Its Luminescent Vapochromic Sensing

Maria Rosaria di Nunzio[1], Mario Gutierrez[1], José María Moreno[2], Avelino Corma[2], Urbano Díaz[2] and Abderrazzak Douhal[1]

[1] Departamento de Química Física, Facultad de Ciencias Ambientales y Bioquímica, and INAMOL, Universidad de Castilla-La Mancha, Avenida Carlos III, S/N, 45071 Toledo, Spain;
mrosaria.dinunzio@uclm.es
mario.gutierrez@uclm.es

[2] Instituto de Tecnología Química, Universitat Politécnica de Valéncia-Consejo Superior de Investigaciones Científicas (UPV-CSIC), Av. de los Naranjos s/n, 46022 Valencia, Spain;
josemmorenorodriguez@gmail.com
udiaz@itq.upv.es
acorma@itq.upv.es

* Correspondence: abderrazzak.douhal@uclm.es; Tel.: +34-925-265717

Received: date; Accepted: date; Published: date

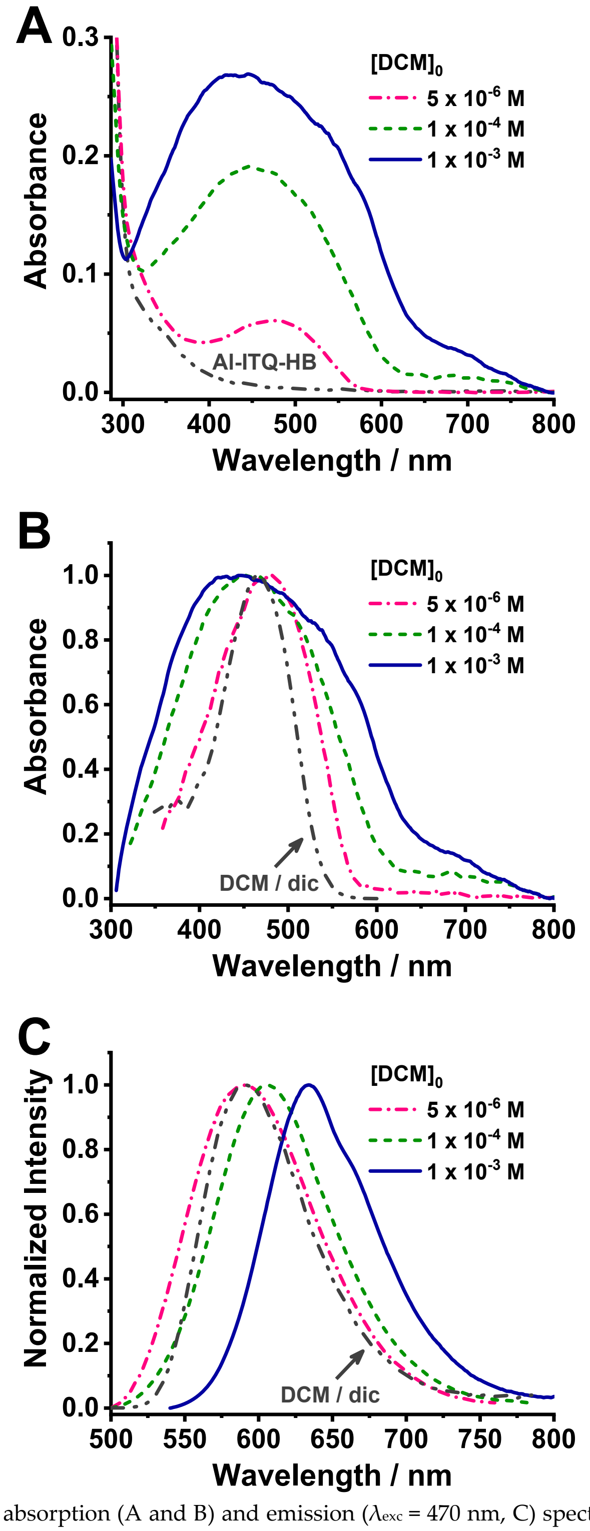

Figure S1. UV-visible absorption (A and B) and emission (λ_{exc} = 470 nm, C) spectra of DCM/Al-ITQ-HB in the solid-state at different initial dye concentrations: 1 × 10^{-3} M (blue solid line), 1 × 10^{-4} M (green dashed line), and 5 × 10^{-6} M (pink dashed-dotted line). In (A), the spectra are not corrected for the pristine Al-ITQ-HB contribution (grey dashed-dotted-dotted line). In (B), each absorption spectrum is obtained by subtracting the pristine Al-ITQ-HB spectrum from the total one. (B) and (C) are normalized (to the maximum intensity) spectra. The absorption (B) and emission (λ_{exc} = 450 nm, C) spectra of DCM in dichloromethane have been added for comparison (grey dashed-dotted-dotted lines).

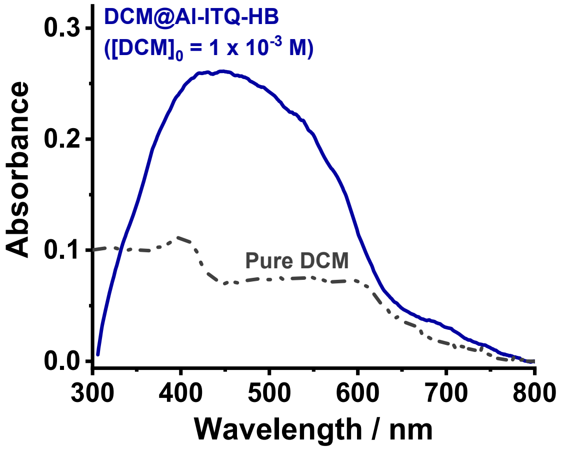

Figure S2. UV-visible absorption spectra of DCM/Al-ITQ-HB ([DCM]$_0$ = 1 × 10^{-3} M, blue solid line) and pristine DCM (grey dashed-dotted-dotted line) in the solid-state.

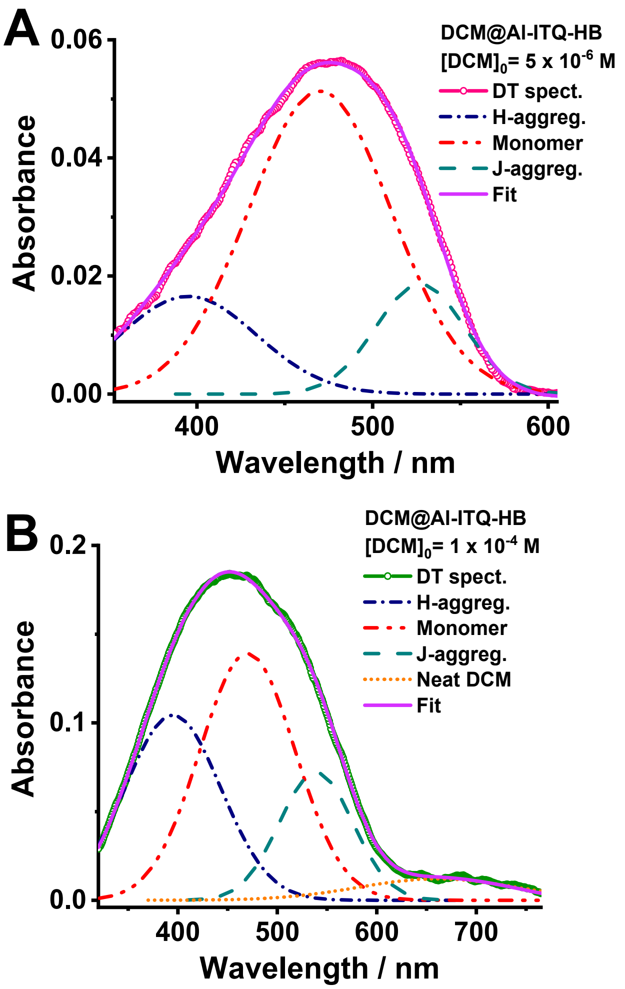

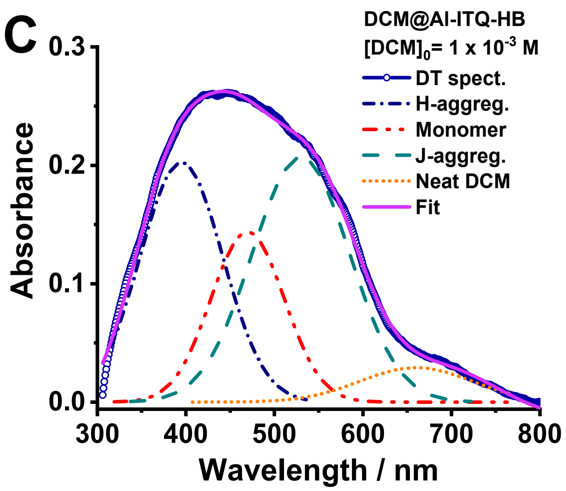

Figure S3. Deconvolution of the UV-visible absorption spectra of DCM/Al-ITQ-HB in the solid-state at different initial DCM concentrations: (A) 5 × 10^{-6} M, (B) 1 × 10^{-4} M, and (C) 1 × 10^{-3} M.

$[DCM]_0$ (M)	H-aggregates		Monomers		J-aggregates		Neat DCM		R_1 (M/H)	R_2 (M/J)	R_3 (H/J)
	λ_{Abs} (nm)	Area (%)	λ_{Abs} (nm)	Area (%)	λ_{Abs} (nm)	Area (%)	λ_{Abs} (nm)	Area (%)			
**5 × 10^{-6}**	395	18	469	67	528	15	-	-	3.7	4.5	1.2
**1 × 10^{-4}**	395	32	470	44	538	18	662	6	1.4	2.4	1.8
**1 × 10^{-3}**	395	32	469	21	529	41	661	6	0.7	0.5	0.8

Table S1. Values of the maximum intensity wavelengths, integral areas (%), and full width at half maximum (FWHM) values (cm^{-1}) observed in the UV-visible absorption spectra of the formed species of DCM upon interaction with Al-ITQ-HB in the solid-state. The different components (H-aggregates; monomers; J-aggregates; neat DCM) were obtained by a spectral deconvolution of the experimental data. The error associated to the wavelength at the maximum absorption intensity (λ_{Abs}) in the deconvolution analysis is about ~ 5 nm. R_1, R_2, and R_3 are the ratios between the integral areas of monomers and H-aggregates (M/H), monomers and J-aggregates (M/J), and H- and J-aggregates (H/J), respectively.

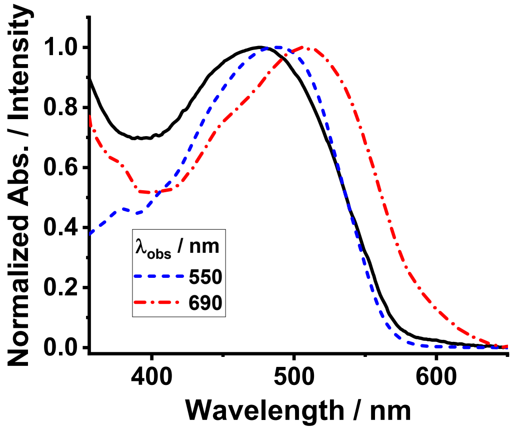

Figure S4. Normalized (to the maximum intensity) UV-visible absorption (black solid line) and excitation (blues dashed and red dashed-dotted lines) spectra of DCM/Al-ITQ-HB in the solid-state with an initial dye concentration of 5×10^{-6} M. The observation wavelengths are listed in the Legend.

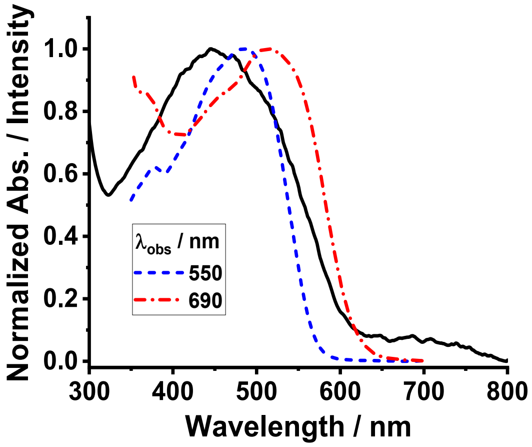

Figure S5. Normalized (to the maximum intensity) UV-visible absorption (black solid line) and excitation (blues dashed and red dashed-dotted lines) spectra of DCM/Al-ITQ-HB in the solid-state with an initial dye concentration of 1×10^{-4} M. The observation wavelengths are listed in the Legend.

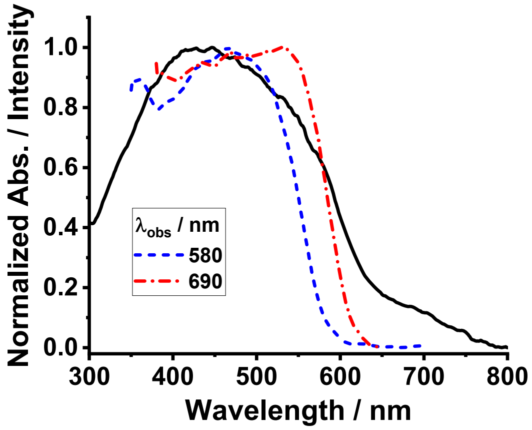

Figure S6. Normalized (to the maximum intensity) UV-visible absorption (black solid line) and excitation (blues dashed and red dashed-dotted lines) spectra of DCM/Al-ITQ-HB in the solid-state with an initial dye concentration of 1×10^{-3} M. The observation wavelengths are listed in the Legend.

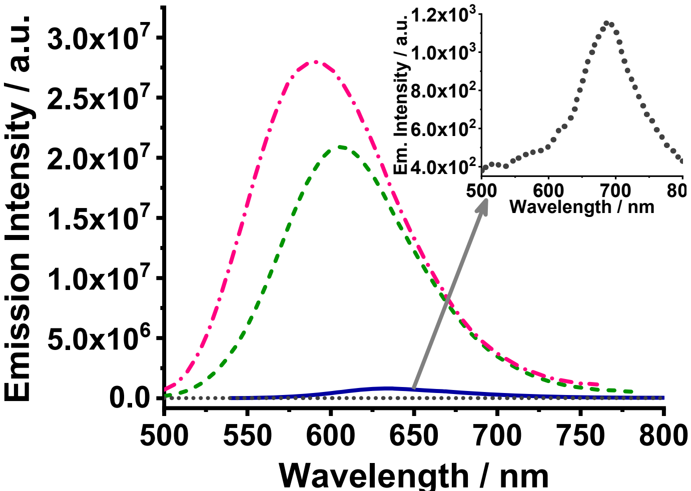

Figure S7. UV-visible emission spectra of DCM/Al-ITQ-HB in the solid-state at different initial dye concentrations: 1×10^{-3} M (blue solid line), 1×10^{-4} M (green dashed line), and 5×10^{-6} M (pink dashed-dotted line). The emission spectrum of DCM solid powder (grey dotted line) is also showed for comparison. The excitation wavelength is 470 nm for all the studied samples.

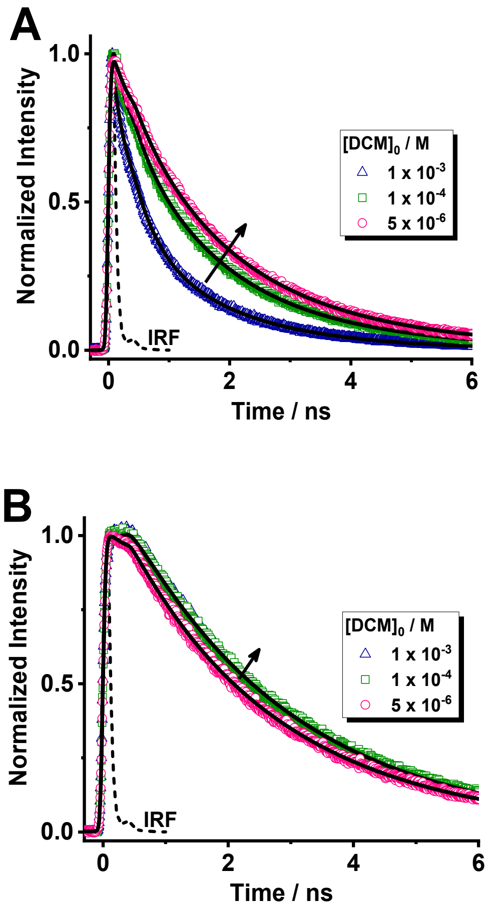

Figure S8. Normalized (to the maximum intensity) magic-angle emission decays of DCM/Al-ITQ-HB at different initial dye concentrations: 1×10^{-3} M (blue triangles), 1×10^{-4} M (green squares), and 5×10^{-6} M (pink circles) upon excitation at 371 nm and observing at 550-575 (A) and 660-750 (B) nm. The solid lines are from the best-fit using a multi-exponential function. IRF (black dashed line) is the instrumental response function.

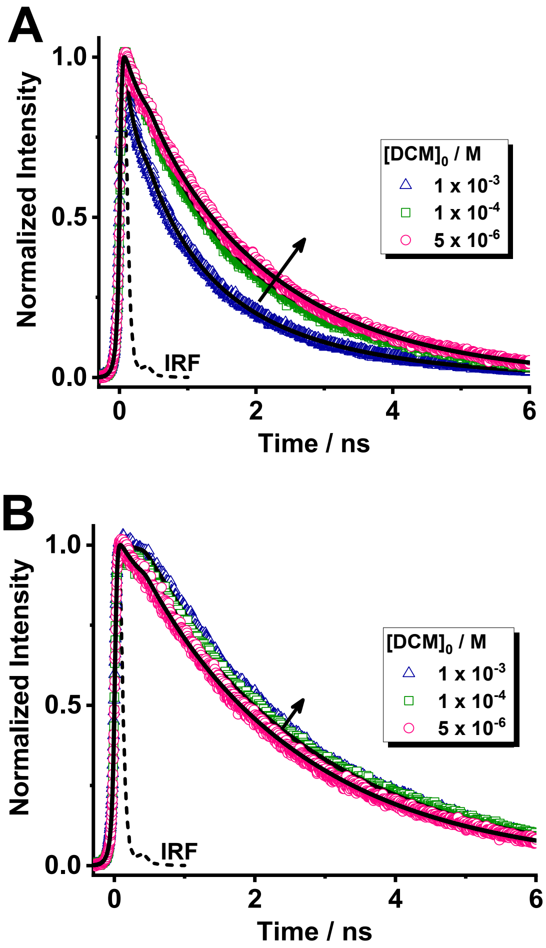

Figure S9. Normalized (to the maximum intensity) magic-angle emission decays of DCM/Al-ITQ-HB at different initial dye concentrations: 1×10^{-3} M (blue triangles), 1×10^{-4} M (green squares), and 5×10^{-6} M (pink circles) upon excitation at 510 nm and observing at 560-575 (A) and 700-750 (B) nm. The solid lines are from the best-fit using a multi-exponential function. IRF (black dashed line) is the instrumental response function.

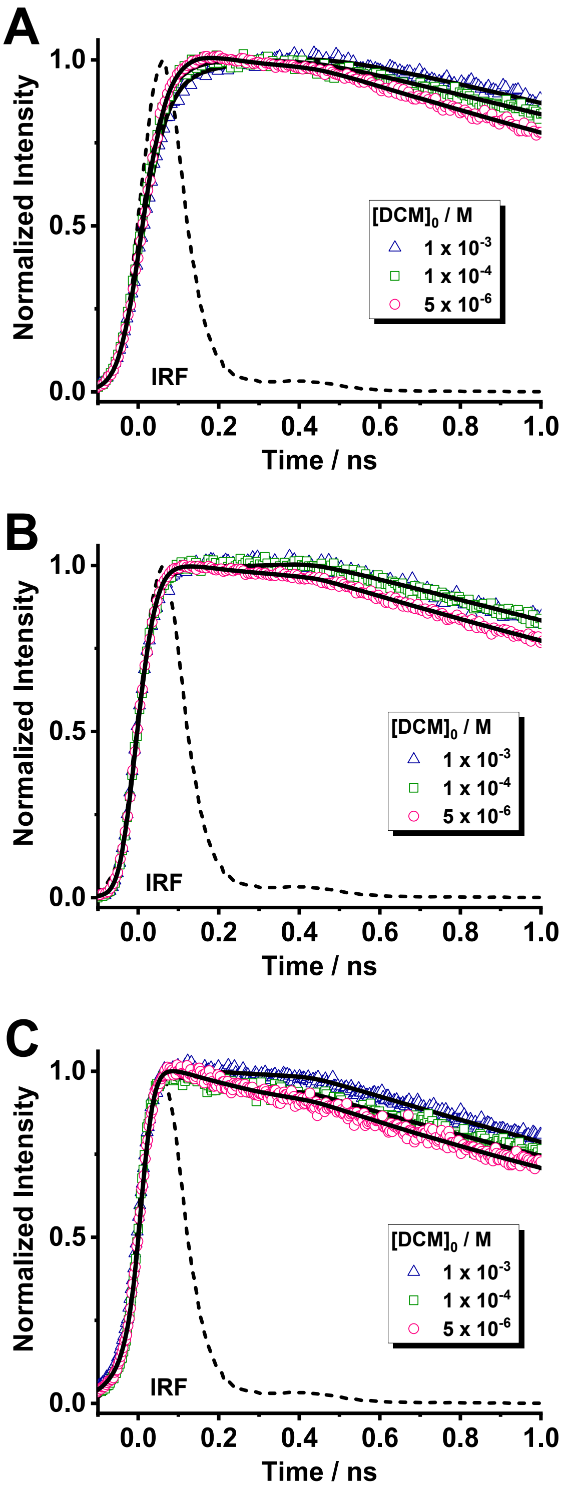

Figure S10. Normalized (to the maximum intensity) magic-angle emission decays of DCM/Al-ITQ-HB at different initial dye concentrations: 1 × 10^{-3} M (blue triangles), 1 × 10^{-4} M (green squares), and 5 × 10^{-6} M (pink circles) upon excitation at 470 (A), 371 (B), and 510 (C) nm and observing at 700-750 (A and C) and 660-750 (B) nm. The solid lines are from the best-fit using a multi-exponential function. IRF (black dashed line) is the instrumental response function.

Sample / Exc = 510 nm	λ_{obs} /nm	τ_1/ps (± 50)	a_1	c_1	τ_2/ns (± 0.2)	a_2	c_2	τ_3/ns (± 0.3)	a_3	c_3
DCM/ Al-ITQ-HB (c_0 = 1 × 10^{-3} M)	560	170	42	7	1.4	51	73	2.7	7	20
	575		34	5		51	61		15	34
	700					18	10		82	90
	720					17	9		83	91
DCM/ Al-ITQ-HB (c_0 = 1 × 10^{-4} M)	560	160	20	2	1.4	53	50	2.7	27	48
	600		7	1		41	28		52	71
	660					19	11		81	89
	700					15	9		85	91
DCM/ Al-ITQ-HB (c_0 = 5 × 10^{-6} M)	560	160	13	1	1.3	42	35	2.4	45	64
	600		8	1		27	19		65	80
	660					11	7		89	93
	700					11	7		89	93

Table S2. Values of time constants (τ_i) and normalized (to 100) pre-exponential factors (a_i) and contributions (c_i) obtained from the fit of the emission decays of DCM interacting with Al-ITQ-HB MOF in the solid-state at different initial dye concentrations: 1 × 10^{-3} M, 1 × 10^{-4} M, and 5 × 10^{-6} M. The excitation wavelength is 510 nm while the observation wavelengths are indicated in the Table. The estimated uncertainty of the time constants, considering the errors from the experiments as well as those arising from the multi-exponential fit of the signals, was between 15 and 20%.

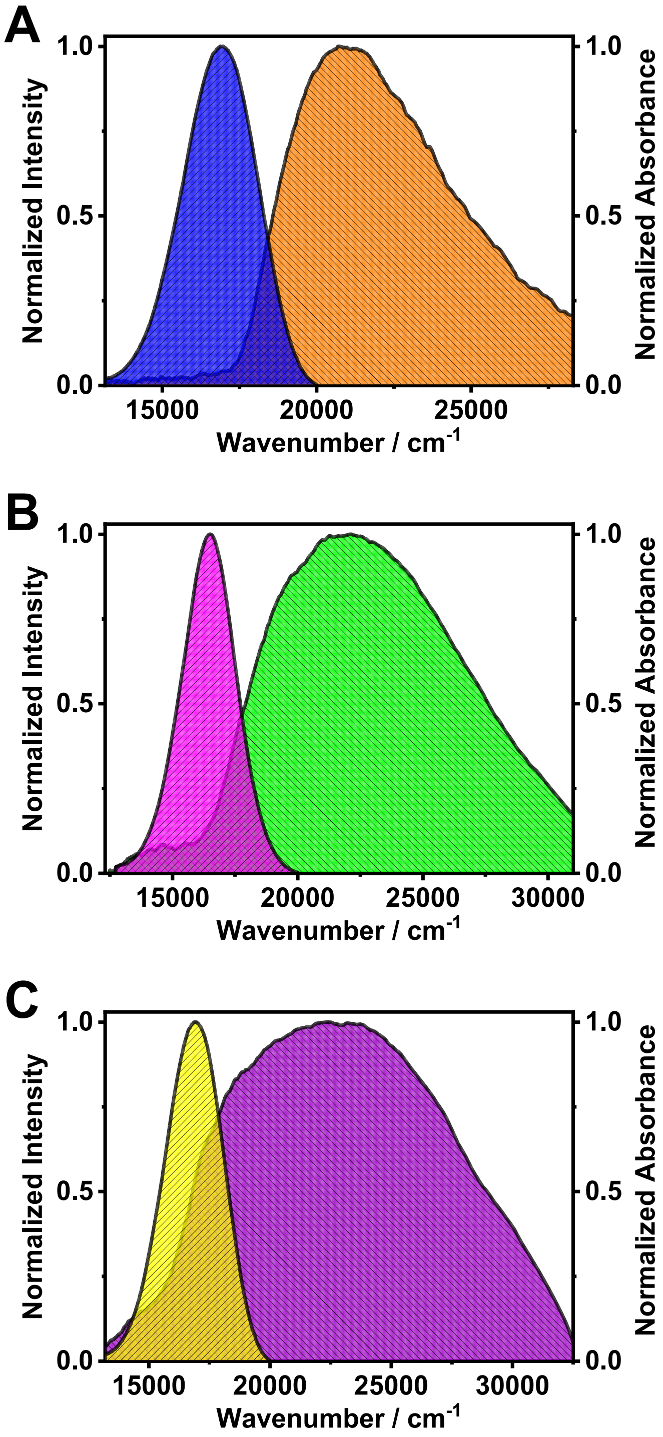

Figure S11. Overlap area between the absorption and emission spectra of DCM/Al-ITQ-HB composite using an initial dye concentration of (A) 5 × 10^{-6}, (B) 1 × 10^{-4}, and (C) 1 × 10^{-3} M.

Characterization

The power X-ray diffraction (PXRD) pattern of the Al-ITQ-HB (HB: heptylbenzoate chain) MOF is reported in **Figure S12**. In particular, a mesoscopic phase was obtained when the monoalkylcarboxylate linker was used as the structural spacer. The PXRD pattern shows one intense *(100)* diffraction band at low 2θ angles range at 35 Å. This characteristic band is associated to mesoporous materials with short-range ordering. This result confirms that mesoporous cavities with high internal diameter are formed for the Al-ITQ-HB material. This phase was found due to the elevated dispersion of the structural 1D-sub-domains because of the presence of dimethylformamide (DMF) as solvent, which are finally organized through mesoscopic morphology. **Scheme S1** describes the mesoscopic structuration level of the hybrid Al-ITQ-HB material.

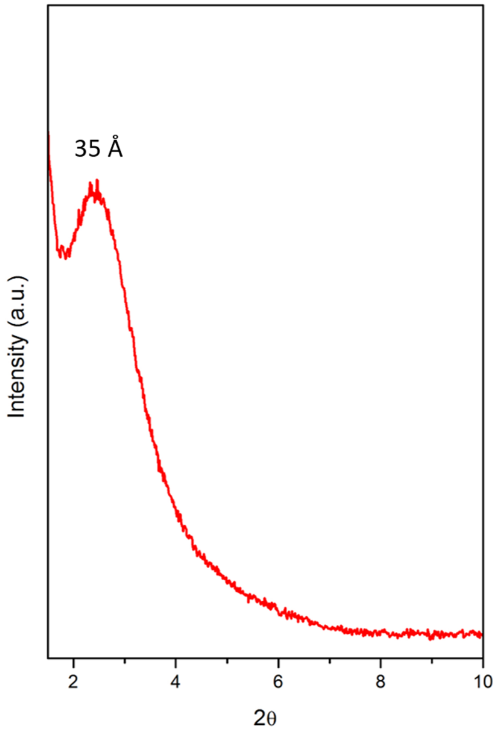

Figure S12. PXRD diffractogram of Al-ITQ-HB (HB: heptylbenzoate chain).

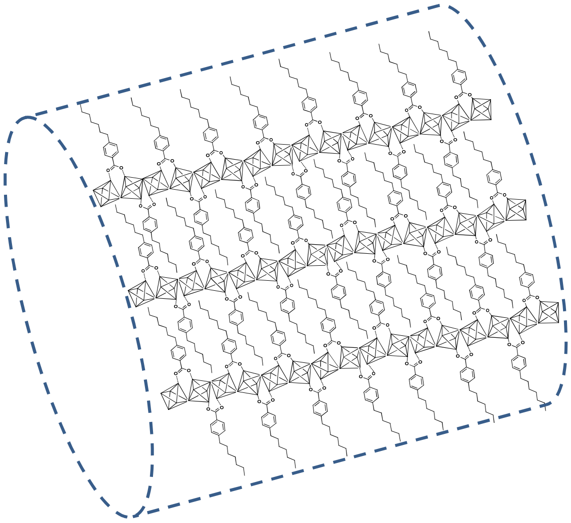

Scheme S1. Structural representation of the Al-ITQ-HB hybrid material.

This type of metal-organic material was synthesized through auto-assembly of specific monotopic organic spacers with aluminium nodes, which were used as builder units to generate hybrid solids exhibiting low-dimensionality based on 1D-organic/inorganic sub-units (**Scheme S1**) under specific conditions through solvothermal synthesis routes. Specifically, heptyl benzene monocarboxylate ligands with *para* conformation were employed as suitable organic molecular spacers to generate this type of 1D structural sub-units (**Scheme S2**).

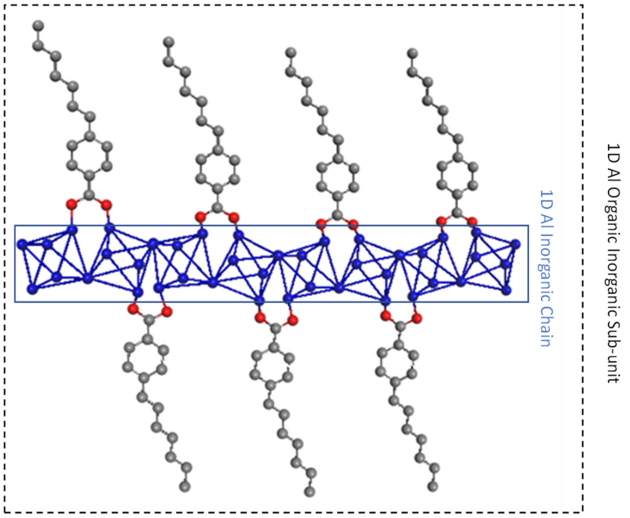

Scheme S2. 1D-organic/inorganic sub-units present in the metal-organic Al-ITQ-HB material.

Elemental CHNS analysis (**Table S3**) of the Al-ITQ-HB material estimated the organic content of the spacers included in the final solids. The organic contribution for Al-ITQ-HB was around 35 wt% due to incorporation of the organic spacer molecules, generating a mesoscopic material with low order structuration and crystallinity. **Table S3** presents low value for nitrogen amount for the mesoscopic material, showing that the DMF molecules used as solvent were totally removed during the solid recovering and washing.

Sample				Org.Cont.[a]		
	C[a]	H[a]	N[a]	CHN[b]	TGA[c]	M[a]
Al-ITQ-HB	34.3	5.3	0.0	39.6	53.1	21.4

[a] Percentage in weight (wt%); [b] Organic content from CHNS elemental analysis, [c] Organic content from thermogravimetric analysis without taking account the hydration water.

Table S3. Elemental analysis of the Al-ITQ-HB material.

Figure S13 (curves of thermogravimetric analysis, TGA; differential thermal analysis, DTA) shows the weight loss for the corresponding organic content in the Al-ITQ-HB material to determine its hydrothermal stability. Two different weight losses were detected without taking account the hydration water and residual DMF molecules occluded in the material (80-150ºC). The first weight loss was observed at 250-400ºC, being associated to the hydrocarbon tails from monocarboxylate organic spacers used for the synthesis of the mesoscopic framework. This weight loss was also assigned to the $AlO_4(OH)_2$ builder units present in the 1D-inorganic chains, being this oxygen contribution the main reason for obtaining the higher organic content from TGA related to CHNS analysis (**Table S3**). This fact indicated that probably that the aluminium nodes were coordinated to elevated number of hydroxylated species. The second weight loss was clearly observed from 450 to 600 °C range corresponding to aromatic fragments from the organic spacers present in the final hybrid structure.

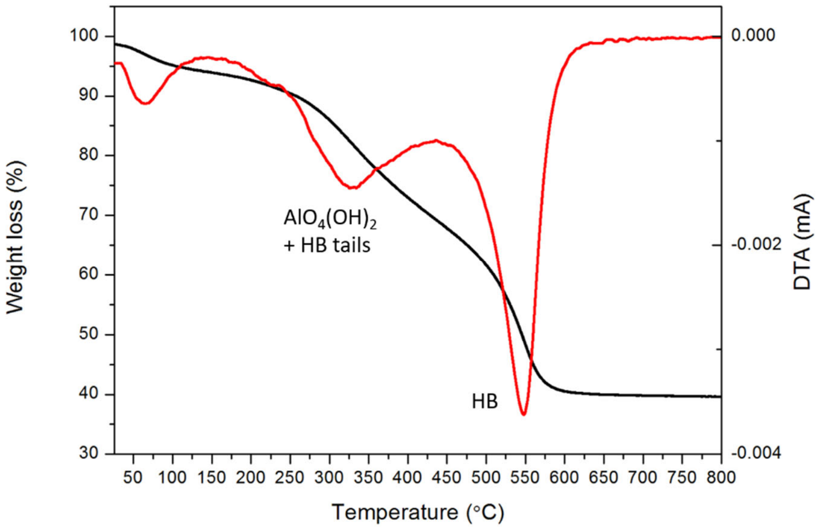

Figure S13. TGA and DTA curves for the Al-ITQ-HB sample.

Micrographs show crystals with poor crystallinity and irregular morphology, indicating low homogeneity and non-ordered structuration (**Figure S14**). In this sample, the mesoscopic long-range order was observed from transmission electron microscopy (TEM). However, the presence of free mesoporous was detected with difficulty due to unstable organic counterpart of the MOF material under the electronic beam. Incorporation of DCM dye in the hybrid material did not imply a substantial modification of the morphology of the solids compared with the pristine Al-ITQ-HB sample (**Figure S15**).

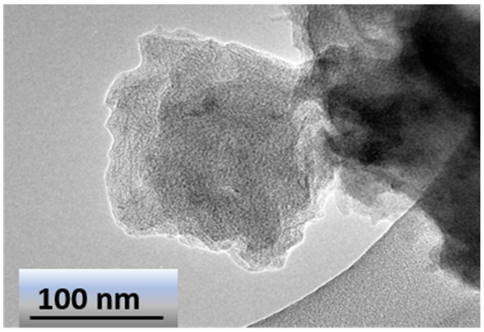

Figure S14. TEM micrograph of the Al-ITQ-HB sample.

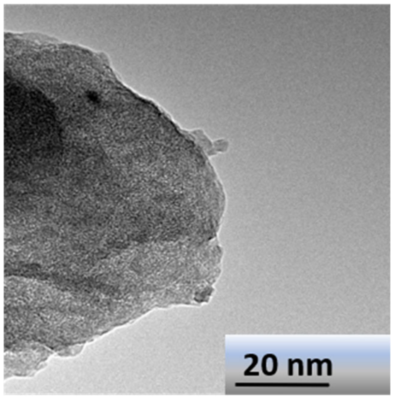

Figure S15. TEM micrograph of the DCM/Al-ITQ-HB sample.

Solid-state cross-polarization magic-angle spinning carbon-13 nuclear magnetic resonance (^{13}C CP/MAS NMR) spectra of the Al-ITQ-HB material is shown in **Figure S16**. A signal at ~ 170 ppm was detected being assigned to the carbon atoms bonded directly to the inorganic chains. At lower chemical shifts, between 110-160 ppm, signals due to the carbon atoms of aromatic rings were found. Moreover, bands associated to -CH_2- from HB alkyl tails were observed in the range of 10-50 ppm. These results confirmed the total integrity of monocarboxylate aromatic organic spacers bonded to the inorganic chains in the hybrid material.

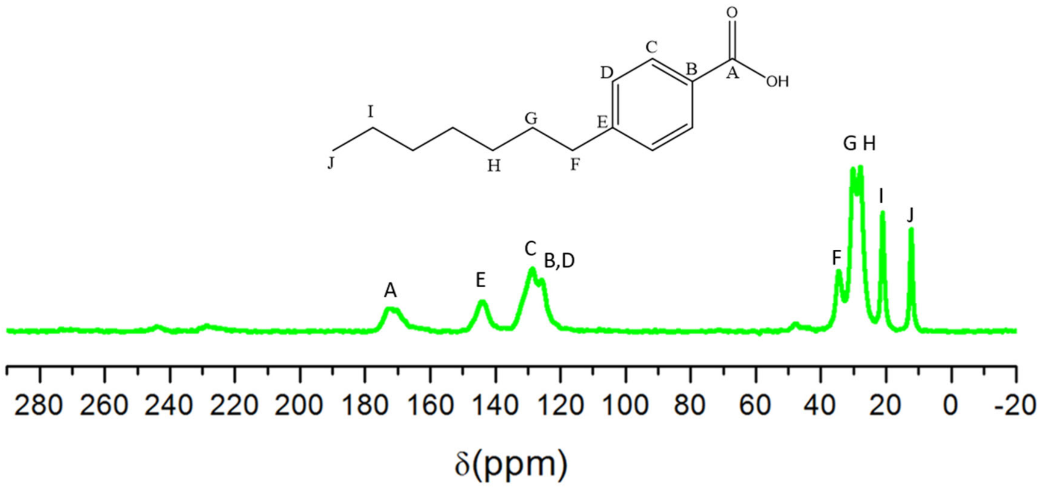

Figure S16. CP/MAS NMR ^{13}C spectrum of Al-ITQ-HB.

It was studied the chemical aluminium environment by solid-state cross-polarization magic-angle spinning aluminium-27 nuclear magnetic resonance (^{27}Al MAS NMR) spectroscopy (**Figure S17**). A chemical shift around 0 ppm was detected, indicating the octahedral position of the aluminium atoms due to the aluminium (oxo)hydroxide species that form the 1D-nanoribbons inorganic chains.

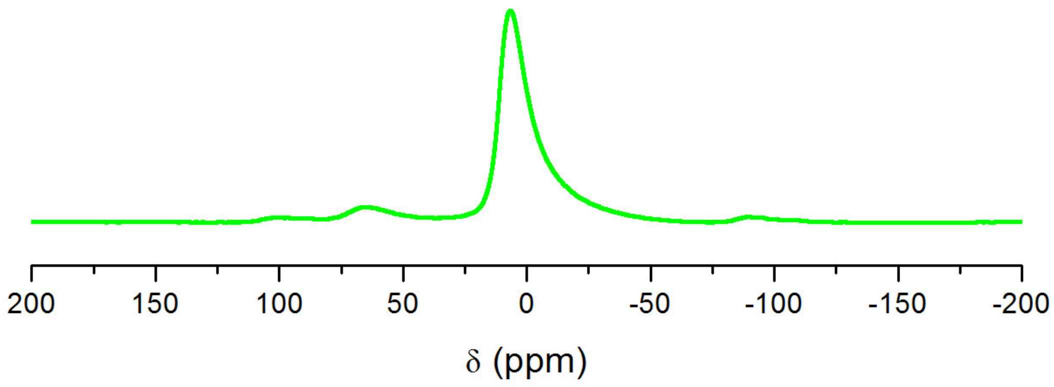

Figure S17. BD/MAS ^{27}Al MAS NMR spectrum of Al-ITQ-HB.
